# Structural and Computational Insights into the Angiotensin II Type 1 Receptor: Advances in Antagonist Design and Implications for Hypertension Therapy (2020–2024)

**DOI:** 10.3390/biom16010020

**Published:** 2025-12-22

**Authors:** Filippos Panteleimon Chatzipieris, Errikos Petsas, George Lambrinidis, John M. Matsoukas, Thomas Mavromoustakos

**Affiliations:** 1 Laboratory of Organic Chemistry, Department of Chemistry, National and Kapodistrian University of Athens, 15771 Athens, Greece; fchatzip@chem.uoa.gr (F.P.C.); errpets@chem.uoa.gr (E.P.); 2 Division of Medicinal Chemistry, Department of Pharmacy, School of Health Sciences, National & Kapodistrian University of Athens, 15771 Athens, Greece; lamprinidis@pharm.uoa.gr; 3 Institute for Health and Sport, Victoria University, Melbourne, VIC 3030, Australia; 4 Department of Physiology and Pharmacology, Cumming School of Medicine, University of Calgary, Calgary, AB T2N 4N1, Canada; 5 NewDrug, P.C., Patras Science Park, 26504 Patras, Greece; 6 Department of Chemistry, University of Patras, 26504 Patras, Greece; imats@upatras.gr

**Keywords:** hypertension (HT), renin–angiotensin–aldosterone system (RAAS), angiotensin II type 1 receptor (AT1R), active site of AT1R, classical pathway of RAAS, alternative pathway of RAAS, Structure-Activity Relationship (SAR), novel AT1R antagonists (2020–2024), sartan derivatives

## Abstract

The renin–angiotensin–aldosterone system (RAAS) is essential for controlling blood pressure and maintaining fluid balance, driving significant structural changes throughout the cardiovascular system, including the heart and blood vessels. As a result, the RAAS is a key therapeutic target for various chronic cardiovascular diseases, ranging from arterial hypertension (AH) to heart failure (HF). In this review, one of our objectives is to describe the new evidence over the last 4 years regarding the RAAS. Moreover, we pay attention to the structure and function of the angiotensin II type 1 receptor (AT1R) and its role in hypertension, as well as define its active site. Later, we discuss the most potent, selective inhibitors of AT1 receptors, based on in vitro and in vivo experiments, from 2020 to 2024. Large peptide molecules, small non-peptide-like molecules, and sartan derivatives are analyzed. The low IC_50_ values of the entities that do not resemble sartans showcase the vast chemical space that can be explored for the creation of more potent antihypertensive medications. We have also employed computational chemistry tools in order to identify key molecular interactions between the compounds of the literature studied in order to elucidate the underlying reasons why these different molecules exhibit variations in their binding energies and overall potency.

## 1. Introduction

Hypertension, or high blood pressure, is a condition where the force of blood pushing against the walls of arteries is consistently too high (≥130/80 mm-Hg in comparison to ≤120/80 mm Hg in healthy individuals) [1]. This condition is associated with many health issues, especially cardiovascular problems such as heart disease, stroke, and heart failure, with cardiovascular disease being the leading cause of mortality worldwide [2]. The fact that hypertension induces vascular and heart disorders, leading to a lot of fatal diseases, justifies its nickname “the silent killer”. Additionally, high blood pressure can impact other organs, causing kidney damage, vision impairment, and vascular dementia [3]. From 2025 to 2050, cardiovascular disease prevalence is expected to rise by 90.0%, with cardiovascular deaths projected to reach 35.6 million in 2050, up from 20.5 million in 2025 [4,5]. Globally, high blood pressure is responsible for roughly 54% of strokes and 47% of coronary heart disease cases [6].

The most common drugs used for hypertension therapy are thiazide-type diuretics, calcium channel blockers, angiotensin-converting enzyme (ACE) inhibitors, and angiotensin II receptor blockers (ARBs) [7]. We focus our attention on molecules that inhibit angiotensin II type 1 (AT1) receptors and, thus, act as ARBs. Thiazide diuretics act as good vasodilators, reducing cardiac output acutely by reducing extracellular fluid (ECF) and plasma volume [8]. Calcium channel blockers prevent calcium influx intracellularly, which is required for myocardial, vascular, and gastrointestinal (GI) smooth muscle contraction, thus leading to vasodilation and hypotension [9]. Major toxicities resulting from thiazide diuretics are hypokalemia, a life-threatening condition that can cause arrhythmias (abnormal heartbeat), hyponatremia, and hyperglycemia [8], while calcium channel blockers may present with life-threatening bradycardia, hypotension, hyperglycemia, and renal insufficiency [9]. ACE inhibitors and ARBs do have side effects, one of major importance being hyperkalemia, with potential life-threatening effects, particularly in patients with chronic renal insufficiency. Up to 10% of patients can develop and experience mild hyperkalemia. That is why serum potassium concentration should be monitored shortly after initiation of therapy with ACE inhibitors and ARBs [10]. The most common side effect of ACE inhibitors is persistent dry coughing due to the accumulation of bradykinin, and a few patients develop angioedema [11]. In severe cases, angioedema can cause swelling in the tongue or throat that blocks the airway, becoming life-threatening [12]. However, it is a rare side effect of both types of drugs [13]. ARBs are generally better tolerated by patients than ACE inhibitors [14] and thus are deemed as more efficient therapies for hypertension [15,16].

Thus, the focus of this review is on the study of novel potent inhibitors of AT1R. Specifically, we examine the newly developed inhibitors from 2020 to 2024 to achieve the aforementioned goals. In this study, large peptide molecules, small non-peptidic compounds, and sartan derivatives are analyzed to better understand their inhibitory potential. Notably, the low IC_50_ values observed for these compounds, which do not share structural similarity with sartans, underscore the huge chemical space that remains to be explored for the rational design of more potent antihypertensive agents. Computational chemistry tools are also employed for the analysis of the interactions of the compounds studied with the AT1 receptor. Finally, a pharmacophore model is proposed to assist medicinal chemists in future drug design and development efforts towards antihypertensive treatment.

### 1.1. Description of the RAAS

**Figure 1:**
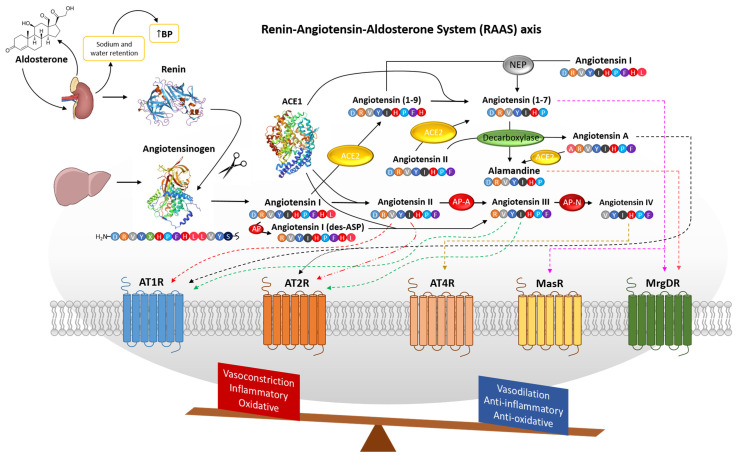
The renin–angiotensin–aldosterone system (RAAS) is of major importance in the regulation of blood pressure and the maintenance of electrolyte balance in the body. Renin is produced in the kidneys when intracellular proteolytic enzymes act on its inactive precursor, prorenin. This enzyme catalyzes the bioconversion of angiotensinogen, an important intermediate protein in angiotensin I (Ang I), the first step in the RAAS pathway. Ang I mainly acts as a precursor for the generation of the other active peptides of the pathway. After the angiotensin-converting enzyme I (ACE1) converts Ang I to angiotensin II (Ang II), Ang II activates AT1R, causing vasoconstriction, triggering inflammation, and leading to oxidative stress. On the contrary, when this peptide interacts with AT2R, it promotes blood vessel dilation, inhibits cell proliferation, and exerts anti-oxidative effects. Aminopeptidase-A (AP-A) converts Ang II to angiotensin III (Ang III), which is then converted to angiotensin IV (Ang IV) with the help of Aminopeptidase-N (AP-N). The end result of this process is the enhancement of cognitive function while also facilitating cellular signal transmission. Angiotensin A (Ang A) is generated from the decarboxylation of Ang II, having both a hypertensive and hypotensive action. Angiotensin 1–9 (Ang 1–9) is generated from Ang I via angiotensin-converting enzyme 2 (ACE2) interacting with AT2R, and it bio-transforms into angiotensin 1–7 (Ang 1–7) through ACE1. Ang I can also be cleaved directly by NEP to form Ang 1–7. All of these bioactive peptides can be created via many different ways, as indicated in the figure and the main body of the review. Through decarboxylation of Ang II, Alamandine (ALA) is created, exerting cardioprotective, anti-inflammatory, and anti-fibrotic effects. Aldosterone, a hormone produced by the adrenal cortex, induces sodium and water retention and increases blood volume and blood pressure. Due to the complexity of the system, where many different parameters can be deregulated, the need to maintain the system in equilibrium becomes unprecedented.

RAAS is a key system in regulating blood pressure and maintaining the balance of water and electrolytes in the body by adjusting plasma sodium concentration and the volume of extracellular fluid. RAAS is made up of interconnected components, including the enzyme renin, which acts as a hormone; angiotensin peptides with diverse functions; and the mineralocorticoid aldosterone [17]. The RAAS can function both systemically and locally. Its systemic role involves regulating arterial blood pressure and sodium levels through the kidneys [18]. It has also been demonstrated that RAAS includes components with an opposing effect, capable of lowering blood pressure through short fragments of angiotensin that counteract the peptides of the classical pathway. These components are referred to as the alternative pathway of RAAS. Angiotensin 1–7 (Ang 1–7), angiotensin 1–9 (Ang 1–9), and alamandine (ALA) are identified as peptides from the alternative pathway that have antihypertensive effects (Figure 1) [19].

### 1.2. Components of RAAS

#### 1.2.1. The Classical Pathway

##### Renin

The activity of renin in the body is essential for the proper functioning of the RAAS. Renin is produced and released by cells in the juxtaglomerular apparatus, which is situated in the afferent arteriole of the kidneys. Renin is generated when intracellular proteolytic enzymes act on its inactive precursor, prorenin [20]. Prorenin is a protein consisting of 406 amino acids that becomes active after undergoing specific processing steps. Activation of prorenin can occur through both proteolytic and non-proteolytic mechanisms. In the proteolytic pathway, prorenin is converted to active renin in the kidneys by enzymes such as neuroendocrine convertase 1 or cathepsin. In contrast, non-proteolytic activation takes place in various tissues through the prorenin/renin receptor, which induces a conformational change in the molecule without cleaving it [21]. Renin facilitates the conversion of angiotensinogen into angiotensin I (Ang I) [22]. Renin expression rises in response to low blood sodium, reduced arterial pressure, or activation of the sympathetic nervous system. Renin cleaves the α-2-globulin angiotensinogen at the leucine–valine bond to generate Ang I (Figure 1). Plasma angiotensinogen levels, in turn, are elevated by thyroid hormones, estrogens, and corticosteroids [21].

##### Angiotensinogen

Angiotensinogen (AGT) is the sole precursor of all angiotensin peptides. It is composed of 485 amino acid residues, including a 33-amino-acid signal sequence [23,24]. AGT is primarily synthesized by hepatocytes and in smaller quantities in the heart, kidneys, brain, adrenal glands, adipocytes, and the blood vessel endothelium, where it is released locally [25]. Immediately after synthesis, the AGT protein is secreted extracellularly. When the ten-amino-acid peptide Ang I is enzymatically cleaved from AGT, the leftover molecule is known as des(Ang I)-AGT [23,26]. Even though des(Ang I)-AGT retains 98% of the original protein’s sequence, its role and biological functions in the body remain unclear. The angiotensin I (Ang Ι) decapeptide (Asp-Arg-Val-Tyr-Ile-His-Pro-Phe-His-Leu) is formed when renin cleaves the N-terminal segment of AGT [27,28,29]. Although it lacks notable biological activity itself, Ang I mainly acts as a precursor for the generation of other active peptides such as angiotensin II (Ang II), angiotensin III (Ang III), angiotensin IV (Ang IV), and angiotensin 1–7 (Ang 1–7) or angiotensin 1–9 (Ang 1–9) (Figure 1) [30].

##### Angiotensin Converting Enzyme Type 1 (ACE1)

ACE1 is an enzyme belonging to the dipeptidase and endoprotease families. Its primary function is to hydrolyze Ang I into Ang II by removing two amino acids from the peptide’s C-terminus (Figure 1). The ACE1 glycoprotein is mainly found attached to the cell membranes of vascular endothelial cells in the lungs—where it is most active—as well as in the proximal renal tubules and neuroepithelial cells. The enzyme’s activity relies on chloride and zinc ions and can be inhibited by chelating agents such as heavy metals, sulfhydryl compounds, and certain peptides. A well-known inhibitor of ACE1 is captopril [31,32]. ACE1 also plays an important role in the degradation of the vasoactive nonapeptide bradykinin [33]. ACE also metabolizes several peptides beyond Ang I and exerts multiple physiological actions, including roles in renal development and male reproduction. In addition, ACE influences both innate and adaptive immunity by modulating macrophage and neutrophil activity. When ACE is overexpressed, these immune effects are amplified—macrophages display enhanced antitumor and antimicrobial functions—while neutrophils show an increased capacity to generate superoxide with potent bactericidal properties [34].

##### Angiotensin II

Angiotensin II (Ang II) (Asp-Arg-Val-Tyr-Ile-His-Pro-Phe) is the most biologically active peptide among the hypertensive angiotensin-related peptides [35]. Its effects result from binding to type 1and type 2 angiotensin II receptors. This octapeptide has a higher affinity for the angiotensin II type 1 receptor (AT1R), predominantly found in the kidneys, vascular smooth muscle, lungs, and liver [36], and a lower affinity for the angiotensin II type 2 receptor (AT2R), which is mainly expressed during the prenatal period and childhood (Figure 1) [37]. In adults, AT2R is primarily present in the kidneys, heart, and blood vessels. Both receptors are part of the G protein-coupled receptor superfamily and have opposing effects. Activation of AT1R can cause vasoconstriction, trigger inflammation, lead to myocardial hypertrophy and fibrosis, and stimulate aldosterone release from the adrenal cortex. In contrast, AT2R activation promotes blood vessel dilation, inhibits cell proliferation and myocyte hypertrophy, and stimulates the release of nitric oxide [38].

##### Aldosterone

Aldosterone, a hormone produced in the zona glomerulosa of the adrenal cortex, controls water and electrolyte balance [39]. It is primarily released in response to elevated extracellular levels of Ang II or potassium [40]. Additional factors related to changes in extracellular fluid (ECF) volume and stress can also modulate its secretion. In humans, aldosterone accounts for roughly 90% of total mineralocorticoid activity. Its main action is the activation of mineralocorticoid receptors (MRs) in principal cells of the distal tubules, cortical collecting ducts, and collecting ducts [41], where it promotes sodium reabsorption and enhances potassium excretion (Figure 1) [42]. The reabsorption of Na^+^ and water is regarded as the primary mechanism underlying the increase in blood pressure associated with activation of the MRs. Research shows that primary aldosteronism is a frequent cause of hypertension, occurring in 5–10% of general hypertension cases and about 20% of patients with severe or treatment-resistant hypertension [43]. The primary action of aldosterone is to induce sodium and water retention and increase blood volume [42]. Excess aldosterone is recognized as a significant cardiovascular risk factor, contributing not only to hypertension but also to stroke, coronary artery disease, heart failure, and diabetes mellitus. Elevated aldosterone levels promote vascular dysfunction and structural remodeling, enhance the production of reactive oxygen species (ROS), and drive inflammatory processes [44,45,46,47,48].

Approximately 10% of individuals with hypertension suffer from treatment-resistant disease, which is defined as persistent elevation of blood pressure despite the use of three antihypertensive agents at appropriate doses—typically a calcium channel blocker, a thiazide diuretic, and either an angiotensin-converting enzyme inhibitor or an angiotensin II receptor blocker (ARB) [49]. The ability of adrenal zona glomerulosa cells to synthesize aldosterone is primarily governed by the regulated transcription of CYP11B2, the gene encoding aldosterone synthase. The resulting enzyme is a member of the cytochrome P450 family, which catalyzes the key rate-limiting step in aldosterone production, aside from cholesterol transport into the mitochondria [50].

Aldosterone synthase inhibition has therefore gained attention as an additional therapeutic strategy alongside MR antagonism. By lowering aldosterone levels in both plasma and tissues, this approach seeks to diminish not only MR-mediated actions but also MR-independent effects across cardiac, vascular, and renal tissues [50]. Specifically, in a randomized clinical trial (Target-HTN) that included 200 participants, lorundrostat, a CYP11B2 inhibitor, decreased blood pressure significantly more than placebo with 50 mg and 100 mg once-daily doses in patients with uncontrolled hypertension despite background antihypertensive treatment [51]. Thus, aldosterone synthase inhibitors can be proven as novel therapies for the treatment of inadequately controlled hypertension.

##### Angiotensin III

Angiotensin III (Ang III) (Arg-Val-Tyr-Ile-His-Pro-Phe) [52] is a peptide produced directly from Ang II through the action of aminopeptidase-A (AP-A) [53]. Another pathway for its formation involves the conversion of Ang I; circulating aminopeptidases first cleave Ang I to Ang I (des-ASP), which is then further broken down into Ang III by ACE1 (Figure 1) [54]. A hypothesis has emerged suggesting that Ang III may be the primary active peptide in the RAAS. This idea is supported by the rapid conversion of Ang II to Ang III, Ang II’s relatively short half-life, and Ang III’s ability to interact with AT1R and AT2R [53]. However, more studies, especially involving humans, are needed to confirm this hypothesis.

##### Angiotensin IV

Recent studies indicate that angiotensin IV (Ang IV) exhibits minimal hypotensive activity but displays significant effects in other physiological systems. It is generated from Ang III through the action of aminopeptidase-N (AP-N) [52] and exerts its functions via the specific AT_4_ receptor (Figure 1). The AT_4_ receptor is broadly distributed across various tissues, including the brain, adrenal glands, kidneys, lungs, and heart. In the kidneys, Ang IV enhances renal cortical blood flow and decreases sodium ion transport within the isolated proximal tubules [55]. The AT_4_ receptor has been identified as a transmembrane enzyme known as insulin-regulated aminopeptidase (IRAP) [56]. IRAP is a type II integral membrane protein from the M1 aminopeptidase family, primarily located in GLUT4 vesicles of insulin-sensitive cells [57]. Activation of the AT_4_ receptor enhances cognitive function [58] and facilitates cellular signal transmission [59,60]. These effects are partly attributed to increased cellular glucose availability mediated by AT_4_. However, Ang IV has only a minimal influence on arterial blood pressure.

##### Aminopeptidase A and N

Both enzymes are membrane-bound zinc metallopeptidases [61,62]. AP-A catalyzes the removal of the N-terminal aspartate from Ang II to produce Ang III, while AP-N subsequently cleaves the N-terminal arginine from Ang III to generate Ang IV (Figure 1) [52]. These peptides are biologically active within the brain, where they contribute to the formation of the local renin–angiotensin system (RAS) [63]. Studies in rats have demonstrated that brain-derived Ang III exerts a more potent hypertensive effect than peripheral Ang II [64]. Consequently, selective inhibitors of AP-A and AP-N are proposed as potential therapeutic agents for hypertension management [65].

##### Angiotensin A

Angiotensin A (Ang A), with sequence Ala-Arg-Val-Tyr-Ile-His-Pro-Phe (differs from Ang II in Ala1 instead of Asp1), exhibits hypertensive effects through the classical pathway and antihypertensive properties, indirectly, via the alternative pathway. Ang A is generated from Ang II through the decarboxylation of Asp1, a reaction catalyzed by human mononuclear lymphocytes (Figure 1) [66]. Ang A is present in the circulation at levels below 20% of those of Ang II [67]. It interacts with both AT1R and AT2R receptors, exhibiting a comparable affinity to Ang II for AT1R, while its affinity for AT2R remains less clearly defined. This peptide occupies an intermediate role between hypertensive and hypotensive actions, as it serves as a substrate for the synthesis of alamandine [68].

#### 1.2.2. The Alternative Pathway

##### Angiotensin 1–7

Angiotensin 1–7 (Ang 1–7) (Asp-Arg-Val-Tyr-Ile-His-Pro) is primarily generated from Ang II through the action of angiotensin-converting enzyme type II (ACE2). Additional enzymes, including angiotensin-converting enzyme type I (via Ang 1–9), proline endopeptidase, neprilysin (NEP), and proline carboxypeptidase (from other angiotensin precursors), also contribute to Ang 1–7 formation. For example, Ang I can also be cleaved directly by NEP to form Ang 1–7 (Figure 1) [30]. Ang 1–7 is the selective endogenous ligand for Mas receptors (MasR). Ang 1–7 lowers arterial blood pressure and counteracts the classical RAAS by promoting vasodilation, stimulating anti-inflammatory prostaglandin synthesis, and enhancing nitric oxide release. Ang 1–7 also benefits the heart by limiting cardiomyocyte proliferation and hypertrophy and promoting coronary vessel regeneration and remodeling. In the kidneys, it regulates sodium transport and enhances glucose reabsorption. Additionally, Ang 1–7 influences lipid metabolism and offers protection against insulin resistance [19,69,70].

##### Angiotensin 1–9

Angiotensin 1–9 (Ang 1–9) (Asp-Arg-Val-Tyr-Ile-His-Pro-Phe-His) is generated from angiotensin I via ACE2 (Figure 1) or tissue-specific enzymes, such as cathepsin A in the heart. Ang 1–9 has been detected in multiple organs, including the heart, kidneys, and testicles. The highest levels of angiotensin 1–9 are found in the endothelium of coronary vessels. Its effects are similar to those of Ang 1–7, though Ang 1–9 exerts a stronger influence on heart tissue than on blood vessels [19,71]. Ang 1–9 signals through the AT2 receptor, and Ang 1–7 can be directly generated from Ang 1–9 via angiotensin-converting enzyme type I (ACE1) and neprilysin (NEP) [72].

##### Alamandine

Alamandine (ALA) and angiotensin 1–7 share almost identical amino acid sequences, differing only at the N-terminal residue (Asp in Ang 1–7 versus Ala in ALA; Ala-Arg-Val-Tyr-Ile-His-Pro). Alamandine is produced either through ACE2-mediated hydrolysis of Ang A or via decarboxylation of the N-terminal Asp in Ang 1–7 (Figure 1) [73]. Unlike Ang 1–7, ALA lacks antiproliferative activity. Despite their structural similarity, ALA exerts its effects through receptors distinct from those targeted by Ang 1–7. To date, ALA is recognized as a ligand for the Mas-related G protein-coupled receptor type D (MrgD), which is expressed in the nervous system—particularly in nociceptive neurons—as well as in muscles, the heart, and testicles [68]. Alamandine has numerous effects, primarily cardioprotective [74], anti-inflammatory, and anti-fibrotic [75], which may help in treating cardiovascular diseases and organ damage [76].

##### Angiotensin Converting Enzyme Type II (ACE2)

ACE2 is a monocarboxypeptidase enzyme expressed on cell membranes, with its catalytic domain facing the circulation to hydrolyze angiotensin peptides. It is predominantly present in the cardiovascular system, intestines, lungs, and kidneys. Within the cardiovascular system, ACE2 is expressed in cardiomyocytes, epicardial adipose tissue, cardiac fibroblasts, smooth muscle cells, and vascular endothelium. The enzyme has two primary functions: it acts as an endogenous regulator of the RAAS and serves as a cellular receptor for SARS-CoV and SARS-CoV-2 viruses [77,78,79].

The discovery of ACE2 has transformed our understanding of blood pressure regulation. The ACE2/Ang 1–7/MasR axis is now considered a counter-regulatory pathway that mitigates the harmful effects of excessive ACE1/Ang II/AT1R activity. ACE2 protects against RAS-induced injury through two processes: (1) degradation of Ang I and Ang II to limit substrate availability on the unfavorable ACE/angiotensin II/AT1R axis and (2) production of Ang 1-7 to increase substrate availability on the protective axis of the ACE2/Ang 1-7/MasR [77,78,79].

### 1.3. Structure and Function of the AT1 Receptor

The human AT1 receptor is composed of 359 amino acids and has a molecular weight of approximately 41 kDa. Similarly, the AT1 receptors in rats and mice also consist of 359 amino acids. It belongs to the G protein-coupled receptors’ superfamily (GPCRs) and exerts an antagonistic effect when compared to the AT2 receptor [80]. Specifically, the activation of the AT1 receptor leads to vasoconstriction, renal salt and water retention, aldosterone, and vasopressin release [81]. On the other hand, when the AT2 receptor is activated, it induces antiproliferative, anti-inflammatory, antifibrotic, vasodilator, and neuroprotective effects [82]. The AT1 receptor shares only 34% sequence identity with the AT2 receptor and rhodopsin [80]. In rodents, two isoforms—AT1A and AT1B—have been identified, sharing over 96% sequence homology and exhibiting identical functions. In contrast, only a single isoform has been identified in humans [83,84]. The AT1 receptor is traditionally known to activate phospholipase C (PLC) through the Gq protein, though it can also interact with Gi, G12/13, and Gs proteins [85].

The AT1 receptor contains three N-glycosylation sites, which facilitate proper receptor folding and membrane trafficking, as well as four cysteine residues located in its extracellular domains [85]. Besides the two cysteine residues that form the characteristic disulfide bridge between the first and second extracellular loops of GPCRs, the AT1 receptor possesses an additional pair of extracellular cysteines. Located in the N-terminal region and the third extracellular loop, these residues create a second disulfide bridge essential for maintaining the receptor’s conformation and facilitating its binding to Ang II [86]. The cytoplasmic region of the AT1 receptor, consisting of three intracellular loops and a C-terminal tail, includes phosphorylation sites for various serine/threonine kinases such as protein kinase C (PKC). It also contains four cysteine residues that participate in the formation of disulfide bridges [85,87].

The AT1 receptor can assume three distinct conformational states that directly modulate its activity [88]. The inactive conformation, stabilized by ARBs, prevents downstream signaling, while the ‘canonical active’ conformation—triggered by binding of the endogenous ligand Ang II—initiates the activation of multiple signaling pathways. Upon Ang II binding, the seventh transmembrane domain shifts on the intracellular side, enabling the recruitment of G proteins or β-arrestin. In contrast, the ‘alternative active’ conformation—characterized by an incomplete movement of this transmembrane domain—prevents G protein coupling to the receptor’s DRY motif and exclusively promotes β-arrestin recruitment and signaling [87,88,89].

The structural requirements of the AT1 receptor vary depending on the process involved, including ligand binding, activation, inhibition, G protein coupling, activation of receptor and non-receptor tyrosine kinases, receptor phosphorylation, and internalization. Ang II interacts with multiple binding sites on the AT1 receptor, located within the extracellular loops (ECLs) and transmembrane helices. The ligand-binding pocket for the hormone is created with amino acid residues of transmembrane helices 2, 3, 4, 5, 6, and 7 [90,91].

### 1.4. AT1 Receptor Active Site

Structure–Activity Relationship (SAR) studies using various Ang II analogs have highlighted the crucial roles of Arg2, Tyr4, His6, Phe8, and the negatively charged carboxy-terminal region in determining the peptide’s biological activity and conformation. Site-directed mutagenesis studies further suggest that residues 1–7 govern receptor affinity, specificity, and signal transduction initiation, while residue 8 is primarily responsible for receptor agonism [91,92].

**Figure 2:**
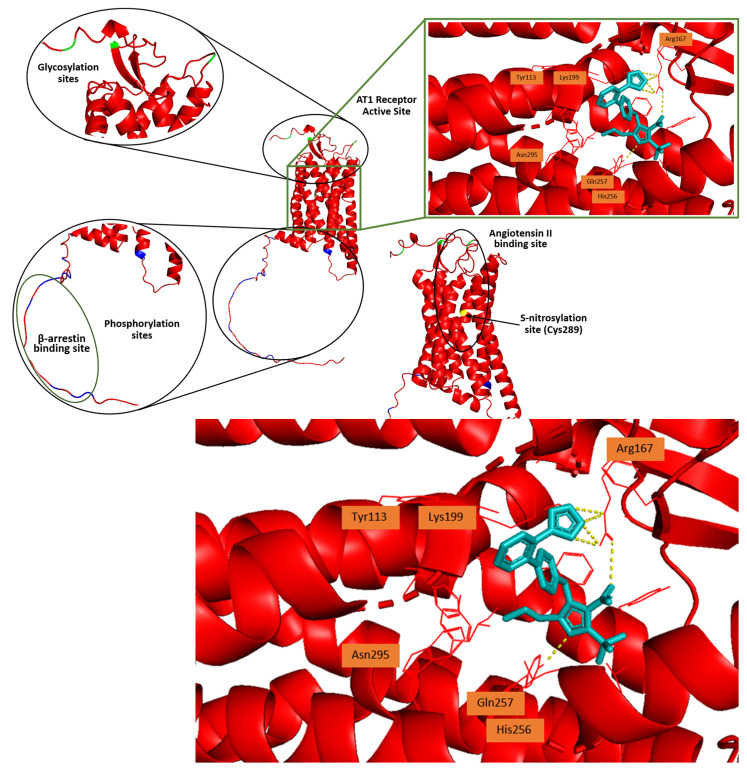
The angiotensin II type 1 receptor (AT1R) showcases its glycosylation (green regions) [97], phosphorylation (blue regions) [98], and S-nitrosylation (Cys289) (yellow region) [99] sites, as well as its active site cavity. The binding sites of β-arrestin and angiotensin II are also presented. In the active site of the AT1R bound with antagonistic drug olmesartan (cyan), the active site’s major amino acid residues can be seen (Tyr113, Lys199, His256, Gln257, Asn295 and Arg167) (*PyMOL*, PDB; 4ZUD).

As previously mentioned, different ligands can induce distinct receptor conformations and stabilize them to varying degrees, influencing agonistic responses. Both peptide and non-peptide antagonists bind to the same AT1 receptor ligand-binding pocket, yet each can produce unique conformational changes on the cytoplasmic side of the receptor that stabilize the bound ligand [93]. Variations in the molecular structure of non-peptide antagonists can influence their binding affinity and the way their functional groups interact with amino acids in the AT1 receptor. For example, irbesartan, which features a cyclopentyl group in place of losartan’s chloride, exhibits the highest affinity, while losartan has the lowest affinity among angiotensin receptor blockers [94]. The binding pocket for non-peptide antagonists is formed by epitopes within the transmembrane domains (TMDs) 3, 4, 5, 6, and 7, with minimal contribution from the extracellular domains. The binding of non-peptide antagonists is critically dependent on Tyr113, Lys199, His256, Gln257, Asn295 and Arg167 to induce inverse agonism toward IP3 (inositol trisphosphate) production (Figure 2) [91,94,95,96].

### 1.5. Artificial Intelligence (AI) in the Drug Design Pipeline

Artificial intelligence (AI) has become an integral part of modern life, driving major advances across a wide range of fields, including image and speech recognition, natural language processing, and more. It essentially refers to the ability of machines to exhibit forms of intelligence [100].

In pharmaceutical research, modern artificial intelligence methods have gained substantial attention, particularly after deep learning architectures achieved outstanding performance in molecular property prediction. Notably, in competitions such as the Merck Kaggle challenge [101] and the NIH Tox21 challenge [102], deep neural networks outperformed traditional machine-learning approaches, demonstrating markedly higher predictive accuracy.

In drug discovery, beyond demonstrating sufficient potency toward the intended biological target, a drug candidate must also show selectivity over off-targets and possess favorable physicochemical characteristics. Additionally, it should exhibit appropriate ADMET properties—absorption, distribution, metabolism, excretion, and toxicity—to ensure its suitability for further development. Within the pharmaceutical industry, extensive data are generated during compound optimization for a wide range of properties. These large target- and antitarget-related datasets, compiled across multiple chemical series, are routinely employed to train machine learning models that support and guide the optimization of new compounds [103].

Deep learning methods have also been utilized for toxicity prediction. Results from the Tox21 challenge demonstrated that deep neural networks (DNNs) achieve strong predictive performance across 12 distinct toxicity endpoints. In this work, particular attention is given to the choice of molecular descriptors alongside physicochemical features and ECFP-style fingerprints; the presence or absence of established toxicophores was incorporated as an additional descriptor set. The study showed that DNNs can automatically extract molecular patterns linked to known toxicophoric motifs, suggesting that these models learn increasingly abstract representations within their hidden layers [102].

De novo design, which seeks to create novel active molecules without relying on existing reference compounds, was introduced roughly 32 years ago. Over the years, many different strategies and software tools have been proposed. However, its adoption in drug discovery has remained limited, in part because the generated structures are often challenging to synthesize. In the last 5 years, advances in artificial intelligence have renewed interest in this approach and revitalized the field [104].

All in all, AI can assist in pharmacodynamic and pharmacokinetic property predictions, as well as de novo drug design. All of these features can greatly assist the work of a medicinal chemist in the drug discovery process.

The aim of AI is to generate putatively more active molecules. In this review, the aim was to discover the most active molecules in the 2020–2024 time period. Based on these molecules, we have performed docking studies in order to reveal any relationship between binding affinity and biological action. The proposed molecules serve as a template for discovering novel molecules. In this aspect, AI is applied to form the pharmacophore, based on the most potent molecules discovered from the literature research, along with some non-potent congener compounds.

## 2. Novel Compounds Found to Be Selective Potent Inhibitors of the AT1 Receptor 2020–2024

A bibliographic search was carried out concerning all molecules that were synthesized from 2020 to 2024 and are deemed selective potent inhibitors of the AT1 receptor. The compounds studied in this review were selected based on their biological activity in vitro and/or in vivo experiments. Thus, compounds that present the lowest IC_50_ values were selected to be presented from each article. Interestingly, the number of articles on the discovery of novel selective AT1 antagonists for the treatment of hypertension is very limited. Specifically, from 2013 to 2024, there were almost no titles for the creation of such inhibitors. The majority of research groups seem to focus on drug repurposing (or repositioning) of already existing antihypertensive drugs, mainly sartans and sartan derivatives, for the treatment of a number of different diseases, ranging from cancer to neurodegenerative disorders, such as Alzheimer’s and Parkinson’s diseases [105]. For instance, Alzheimer’s disease has been mainly studied through the scope of amyloid plaque formation [106], while its cerebrovascular aspect was frequently overlooked. That is why most researchers emphasize their work towards ARB repurposing [105]. Nevertheless, we shall examine these few instances. Structure-Activity Relationship (SAR) studies were also conducted by our research group, and the types of interactions between the various pharmacophore groups of the inhibitors and the respective amino acids of the AT1R are outlined as well.

In order to compare the in vitro results of the following reviewed molecules with a compound of reference, we decided to compare their IC_50_ values with the well-known and established AT1R inhibitor, losartan. Thus, we utilized the study of Burnier et al. (1997) [107] towards this goal. In the study, the early development of AT1 receptor antagonists is described, with losartan being the first clinically available agent. Losartan showed selective AT1 blockade with an IC_50_ of 20 nM (Ki = 10.5 ± 1.2 nM) (Table 1) in rat vascular smooth muscle cells, but its pharmacological profile is largely attributed to its active metabolite EXP3174, which is about 20-fold more potent. Radioligand binding assays and vascular contractility studies confirmed surmountable antagonism by losartan, while EXP3174 induced insurmountable inhibition of Ang II responses. In preclinical models, losartan effectively reduced blood pressure and proteinuria, with efficacy comparable to ACE inhibitors. The IC_50_ value places losartan as a moderate reference antagonist, providing a benchmark for the design of more potent derivatives and subsequent clinical ARBs [107].

In the time period from 2020 to 2024, few are the research papers focused on selective AT1R inhibition. However, these few articles found in the literature can pave new ways for the creation of novel antihypertensive drugs. Liang et al. (2021) [108] utilized DNA-encoded chemical libraries (DECLs), derived from natural product scaffolds, to discover novel AT1 antagonists. The library included 32,000 phenolic acid derivatives, which were screened against immobilized AT_1_ receptors. From this screen, **Hit 1** emerged with an IC_50_ of 19.6 nM (Ki = 0.49 ± 0.03 nM) (Table 1) in [^125^I]-Sar^1^-Ang II competition binding assays. In vivo studies further demonstrated that **Hit 1** significantly lowered blood pressure in renovascular hypertensive rats without affecting heart rate, indicating favorable cardiovascular selectivity [108]. The comparable potency of **Hit 1** to losartan suggests that DECL-based approaches can identify clinically relevant antagonists with efficient screening, positioning **Hit 1** as a promising early-stage lead with IC_50_ values in the same range as the first-generation of ARBs. It should be noted that this is a case of a molecule with a structure not closely related to angiotensin II receptor blockers (ARBs). Instead, it possesses peptide-like characteristics, and it has a relatively large molecular structure.

Wu et al. (2020) [109] synthesized a series of fluoro-substituted benzimidazole derivatives, utilizing radioligand binding and in vivo pharmacological assays in order to confirm their antihypertensive potential. Synthesized compound **1a** is a derivative of valsartan, while compound **1g** is a derivative of telmisartan. In competitive binding assays using vascular smooth muscle cells, compound **1a** displayed an IC_50_ of 8.1 ± 2.4 nM (Ki = 5.8 ± 2.3 nM), while compound **1g** was markedly more potent at 0.8 ± 0.1 nM (Ki = 0.6 ± 0.2 nM) (Table 1). In spontaneously hypertensive rats, both compounds produced significant reductions in mean blood pressure lasting over 24 h, with compound **1g** showing superior activity compared to losartan and telmisartan. These findings indicate that fluorine substitution enhanced hydrophobic interactions with the AT1 receptor, producing subnanomolar antagonists [109]. Compared to the other compounds, losartan and **Hit 1**, the IC_50_ values of **1a** and particularly **1g** represent a substantial improvement in receptor affinity. Compounds **1a** and **1g** are examples where further derivatization of already established ARBs can lead to molecules with even greater activity.

Danilenko et al. (2023) [110] focused on the design and synthesis of indole-3-carboxylic acid derivatives as AT1 receptor antagonists. Radioligand binding studies in vascular smooth muscle cells using [^125^I]-Ang II demonstrated nanomolar affinities for several analogs. Notably, compounds **4a** and **4g** showed IC_50_ values of 11.3 ± 1.2 nM (Ki = 10.8 ± 1.4 nM) and 12.3 ± 1.6 nM (Ki = 11.0 ± 1.5 nM) (Table 1), respectively, comparable to losartan. In vivo experiments in spontaneously hypertensive rats revealed that these compounds significantly reduced blood pressure after oral administration, with maximal decreases of up to 48 mmHg at 10 mg/kg, exceeding the effect of losartan [110]. While not as potent as compound **1g**, the indole-based analogs nonetheless provide valuable scaffolds with balanced receptor affinity and antihypertensive efficacy. This is one of the few times in the literature where small non-peptide molecules showcase such potent inhibition of the AT1 receptor.

**Table 1:** Comparative data of selected AT1 receptor antagonists, presenting their inhibitory activity (IC_50_), their inhibition constants (Ki), their half-life (t_1/2_), and corresponding molecular docking scores.

Name of the Compound	Structure	IC_50_ (nM)	Ki (nM)	Docking Score (kcal/mol)
**Losartan** [107] (t_1/2, human_: ~2 h) [111]	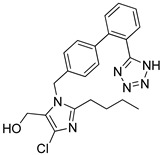	20	10.5 ± 1.2	−9.739
*Group A; large peptide-like molecules*				
**Hit 1** [108] (t_1/2, rat_: ~2.5 h)	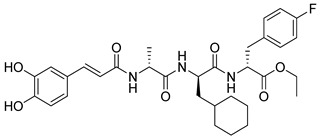	19.6	0.49 ± 0.03	−14.650
*Group B; small non-peptide molecules*				
**4g** [110] (t_1/2, rat_: ~19.9 h)	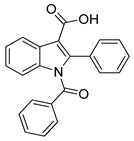	12.3 ± 1.6	11.0 ± 1.5	−10,430
**4a** [110]	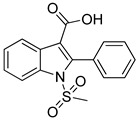	11.3 ± 1.2	10.8 ± 1.4	−10.068
*Group C; sartan derivatives*				
**1a** [109]	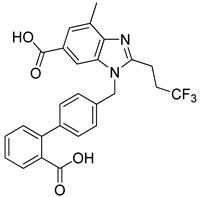	8.1 ± 2.4	5.8 ± 2.3	−14.782
**1g** [109]	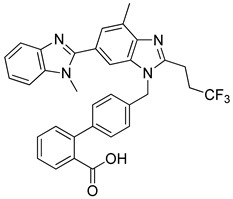	0.8 ± 0.1	0.6 ± 0.2	−12.894
*Group D; super—sartans/bisartans*				
**BisA** [112]	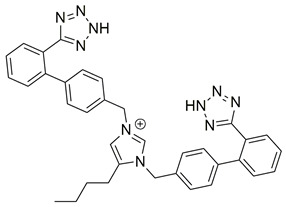	0.35	–	−10.057
**BisB** [112]	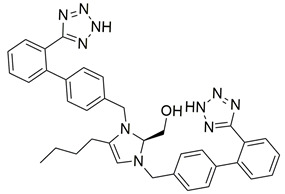	4.27	–	−14.853

## 3. A Different Approach to AT1R Inhibition: The Bisartans’ Case

Another class of molecules, although synthesized before 2020–2024, continues to exert not only antihypertensive properties but also antiviral properties [113]. This can be classified as a fourth group, the “super-sartan” group, or “bisartans”, as they possess a unique chemical structure (combining two sartans in one molecular entity) [112]. These molecules contain two biphenyltetrazole groups, enabling bivalent interactions with critical receptor-based positively charged groups, such as Arg167 of the angiotensin AT1 receptor [114]. Our laboratory has developed and is still synthesizing new molecules, assessing their affinity to the AT1R. A lot of these molecules have shown in vitro higher affinity for the AT1R in comparison to losartan, in the nanomolar range [112,114,115,116,117]. Specifically, compounds **BisA** (also named BV6 or compound **11**) and **BisB** (also named compound **14**) showcase higher antagonistic activity (potency) when compared to losartan [105,112] (Table 1). In silico studies have also demonstrated their abilities as multifunctional agents for the treatment of SARS-CoV-2 infection, influenza, and respiratory syncytial viruses, rendering them as potential pan-antiviral drugs [113,115,118]. For further information on these molecules and representative examples of our work on the AT1R, you can refer to [119,120,121,122,123,124,125,126].

## 4. Computational Experiments

All the above-referenced molecules were docked by our research team on the angiotensin II type 1 receptor (AT1R) (PDB ID: 4ZUD), and we have calculated their binding energies using the computational software Maestro 2021-2 [127]. The lowest energy and most stable ligand–receptor complex conformations were chosen for the presentation and analysis of each of the compounds studied. The ligand interaction diagrams are also shown below.

### 4.1. Molecular Docking Illustrations and Interactions’ Diagrams

**Figure 3:**
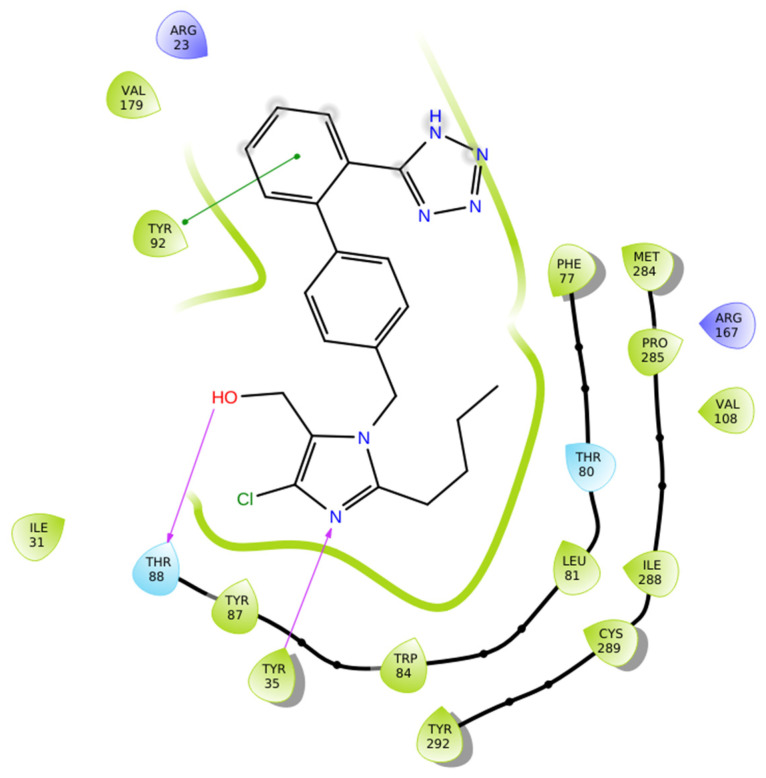
Two-dimensional ligand interactions’ diagram of losartan bound to the AT1 receptor (PDB ID: 4ZUD). Losartan forms hydrogen bonds with Thr88 (–OH) and Tyr35 (imidazole–N) and a pi–pi stacking interaction with Tyr92 (phenyl ring). Hydrophobic contacts are established with Val179, Tyr92, Ile31, Tyr87, Tyr35, Trp84, Leu81, Phe77, Met284, Pro285, Val108, Ile288, Cys289, and Tyr292. Polar interactions involve Thr88, while Arg23 and Arg167 are located as positively charged residues within the binding site. The docking score was −9.739 kcal/mol.

The docking simulation of losartan within the AT1 receptor binding site (PDB ID: 4ZUD) demonstrated a well-defined interactions’ network consistent with its established pharmacological profile (Figure 3). The ligand engages in two key hydrogen bonds, one between the hydroxyl group and Thr88 and another involving the imidazole nitrogen and Tyr35, complemented by a pi–pi stacking interaction between Tyr92 and the phenyl ring, which is directly connected to the tetrazole moiety. The latter enhances aromatic stabilization. Numerous hydrophobic residues (Val179, Tyr92, Ile31, Tyr87, Tyr35, Trp84, Leu81, Phe77, Met284, Pro285, Val108, Ile288, Cys289, and Tyr292) further support the stability of the complex through van der Waals interactions. Polar and charged amino acids (Thr88, Thr80, Arg23, and Arg167) define the electrostatic environment of the binding pocket, contributing to the overall binding orientation (Figure 3). The docking score, −9.739 kcal/mol, reflects a favorable interaction and a stable complex formation. The observed interaction’ network is comparatively moderate, suggesting potential for enhanced binding through structural optimization or alternative ligand scaffolds.

**Figure 4:**
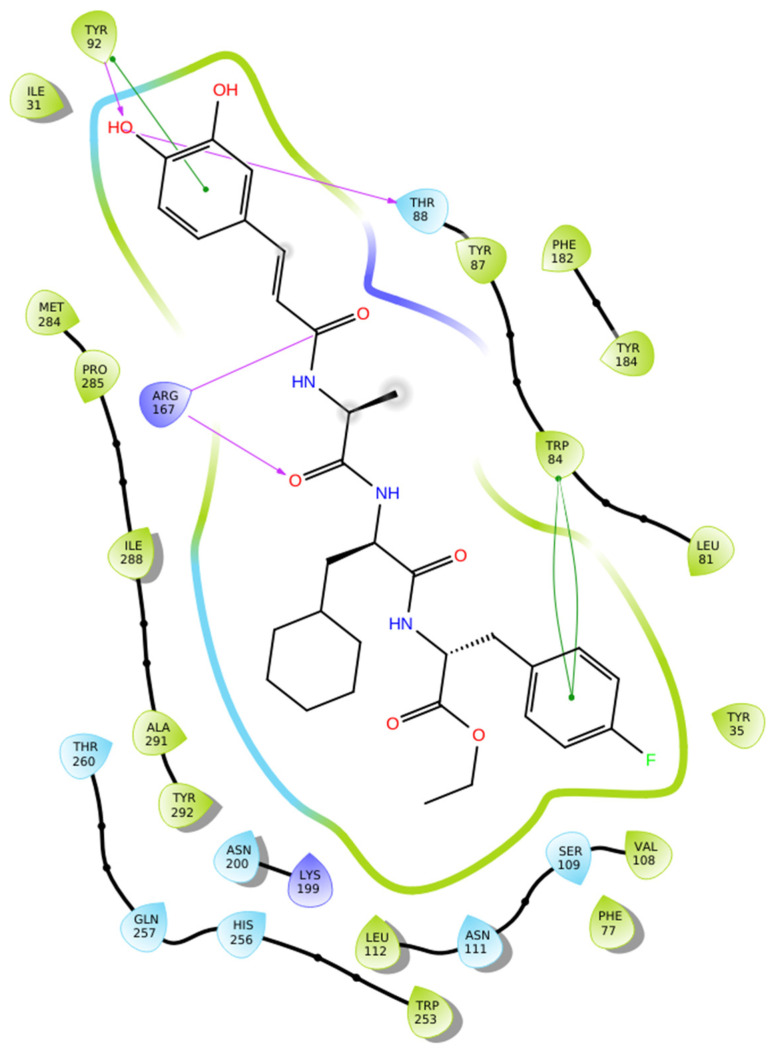
Two-dimensional ligand interactions’ diagram of **Hit 1** bound to the AT1 receptor (PDB ID: 4ZUD). The ligand forms hydrogen bonds between its phenolic hydroxyl group and residues Thr88 and Tyr92, while Arg167 interacts with two carbonyl moieties of amide groups through hydrogen bonding. pi–pi stacking interactions are observed between the phenyl ring of the phenol group and Tyr92. More pi–pi stacking interactions can be observed, also, between the phenyl ring of the aryl fluoride group and the amino acid residue Trp84. Hydrophobic contacts involve Ile31, Tyr92, Tyr87, Trp84, Leu82, Phe182, Tyr184, Met284, Pro285, Ile288, Ala291, Tyr292, Leu112, Val108, Phe77, and Trp253. Polar residues Thr260, Gln257, His256, Asn200, Asn111, Ser109, and Thr88 contribute to the stabilization of the binding environment. Additionally, the positively charged amino acids Arg167 and Lys199 enhance electrostatic stabilization within the binding site. Docking score: −14.650 kcal/mol.

Docking analysis of **Hit 1** within the AT1 receptor (PDB ID: 4ZUD) revealed an extensive interactions’ network that supports a high-affinity ligand–receptor complex (Figure 4). The hydroxyl group of the phenyl ring forms two key hydrogen bonds with Thr88 and Tyr92, while Arg167 interacts via hydrogen bonding with two carbonyl moieties of amide groups, an interaction absent in the losartan–AT1 complex. Furthermore, pi–pi stacking between the aromatic core of **Hit 1** and residues Tyr92 and Trp84 enhances pi-system stabilization within the hydrophobic cavity. A dense array of hydrophobic residues, including Ile31, Tyr92, Tyr87, Trp84, Leu82, Phe182, Tyr184, Met284, Pro285, Ile288, Ala291, Tyr292, Leu112, Val108, Phe77, and Trp253, reinforce the structural complementarity of the complex. Additional polar interactions with Thr260, Gln257, His256, Asn200, Asn111, Ser109, and Thr88 further contribute to the conformational stability of the bound ligand. Compared to losartan, which exhibited a moderate interaction profile (−9.739 kcal/mol) primarily stabilized through limited hydrogen bonding and aromatic stacking, **Hit 1** demonstrates a more intricate and energetically favorable binding pattern (Figure 4). The presence of multiple hydrogen bonds, pi–pi stacking, and hydrophobic and electrostatic interactions collectively results in a significantly improved docking score (−14.650 kcal/mol), indicating a stronger and more stable association with the receptor’s active site.

**Figure 5:**
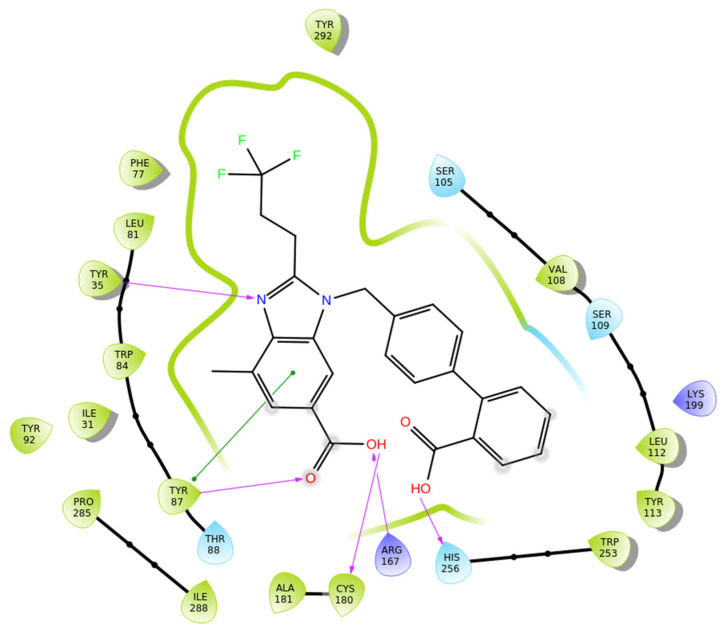
Two-dimensional ligand interactions’ diagram of compound **1a** bound to the AT1 receptor (PDB ID: 4ZUD). The ligand forms hydrogen bonds between the benzimidazole nitrogen and Tyr35, as well as between the carbonyl oxygen of the carboxyl group of the benzimidazole moiety and Tyr87. Additional hydrogen bonds are established between the hydroxyl group of the same carboxyl substituent and Arg167 and Cys180, while His256 interacts with the hydroxyl part of the carboxyl group of the biphenyl moiety. A pi–pi stacking interaction occurs between the phenyl ring of the benzimidazole moiety and Tyr87. Hydrophobic residues involved include Tyr292, Phe77, Leu81, Tyr35, Trp84, Ile31, Tyr92, Pro285, Ile288, Tyr87, Ala181, Cys180, Trp253, Tyr113, Leu112, and Val108. Polar interactions are noted with Thr88, His256, Ser105, and Ser109. The positively charged amino acids Arg167 and Lys199 enhance electrostatic stabilization within the binding site. The docking score (−14.782 kcal/mol) indicates a highly stable and favorable binding conformation.

Compound **1a** exhibited a highly favorable binding mode within the AT1 receptor (PDB ID: 4ZUD), characterized by an extensive network of stabilizing interactions (Figure 5). The benzimidazole–nitrogen forms a hydrogen bond with Tyr35, while the carbonyl–oxygen of the carboxyl group of the benzimidazole–moiety interacts with Tyr87. The same –COOH group establishes additional hydrogen bonds with Arg167 and Cys80, highlighting a multifocal anchoring effect within the binding cavity. His256 contributed further stabilization through hydrogen bonding with the hydroxyl part of the –COOH group of the biphenyl moiety. Moreover, pi–pi stacking between the phenyl ring of the benzimidazole moiety and Tyr87 enhanced the conformational rigidity of the complex. The hydrophobic environment surrounding the ligand, defined by Tyr292, Phe77, Leu81, Tyr35, Trp84, Ile31, Tyr92, Pro285, Ile288, Tyr87, Ala181, Cys180, Trp253, Tyr113, Leu112, and Val108, provides substantial van der Waals stabilization, while polar residues (Thr88, His256, Ser105, and Ser109) contribute to orientation and solvation balance (Figure 5). Compared with losartan (−9.739 kcal/mol) and **Hit 1** (−14.650 kcal/mol), compound **1a** displays a slightly more favorable docking score (−14.782 kcal/mol) and a richer hydrogen bonding network. While losartan engages mainly through limited hydrogen bonds and pi–pi interactions, and **Hit 1** introduces additional aromatic stacking contributions, compound **1a** achieves optimal binding complementarity by combining multiple hydrogen bonds, pi–pi stacking, and extensive hydrophobic contacts. This suggests that **1a** interacts with the AT1 receptor more tightly and with enhanced geometric compatibility compared to both compounds.

**Figure 6:**
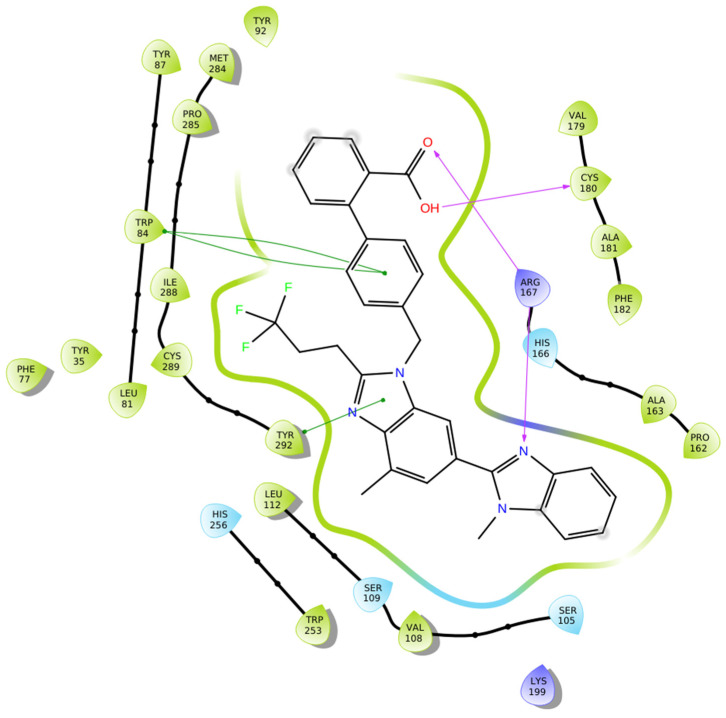
Two-dimensional ligand interactions’ diagram of compound **1g** bound to the AT1 receptor (PDB ID: 4ZUD). Arg167 forms hydrogen bonds with both the carbonyl oxygen of the carboxyl group of the biphenyl moiety and the benzimidazole nitrogen, while the hydroxyl part of the same carboxyl group interacts with Cys180. pi–pi stacking interactions are observed between Trp84 and the benzyl ring of the biphenyl group and between Tyr292 and the imidazole ring of the benzimidazole moiety. Hydrophobic residues include Tyr292, Phe77, Leu81, Tyr35, Trp84, Tyr92, Pro285, Ile288, Tyr87, Ala181, Cys180, Trp253, Leu112, Val108, Cys289, Tyr292, Pro162, Ala163, and Val108. Polar residues His256, Ser105, Ser109, and His166 contribute to the stabilization of the ligand–receptor complex. The positively charged amino acids Arg167 and Lys199 enhance electrostatic stabilization within the binding site. The docking score (−12.894 kcal/mol) indicates a favorable and stable binding conformation.

Compound **1g** demonstrates a strong and well-defined binding profile within the AT1 receptor, characterized by multiple stabilizing interactions that ensure an energetically favorable complex (Figure 6). The carbonyl oxygen of the carboxyl group of the biphenyl moiety forms a hydrogen bond with Arg167, which also interacts with the benzimidazole nitrogen, establishing a dual anchoring effect that enhances the ligand positional stability. The hydroxyl part of the same carboxyl group interacts with Cys180, reinforcing the hydrogen-bonding network within the binding cavity. Furthermore, pi–pi stacking interactions are observed between Trp84 and the ligand’s benzyl ring of the biphenyl group, as well as between Tyr292 and the imidazole ring of the benzimidazole moiety, contributing to aromatic stabilization. The surrounding hydrophobic residues (Tyr292, Phe77, Leu81, Tyr35, Trp84, Tyr92, Pro285, Ile288, Tyr87, Ala181, Cys180, Trp253, Leu112, Val108, Cys289, Tyr292, Pro162, and Ala163) form a compact nonpolar environment that supports van der Waals stabilization, while polar residues such as His256, Ser105, Ser109, and His166 contribute to additional ligand orientation and solvation stability (Figure 6). Although compound **1g** exhibits a slightly less favorable docking score (−12.894 kcal/mol) than compounds **1a** (−14.782 kcal/mol) and **Hit 1** (−14.650 kcal/mol), it still displays a broader interactions’ network and improved electronic complementarity compared with losartan (−9.739 kcal/mol). The dual hydrogen bonding with Arg167, combined with extensive hydrophobic and pi–pi interactions, reflects a well-optimized binding orientation. These results suggest that while **1g** maintains a strong affinity toward AT1R, further structural refinement might enhance its binding energy and receptor complementarity relative to the top-performing analogs **1a** and **Hit 1**.

**Figure 7:**
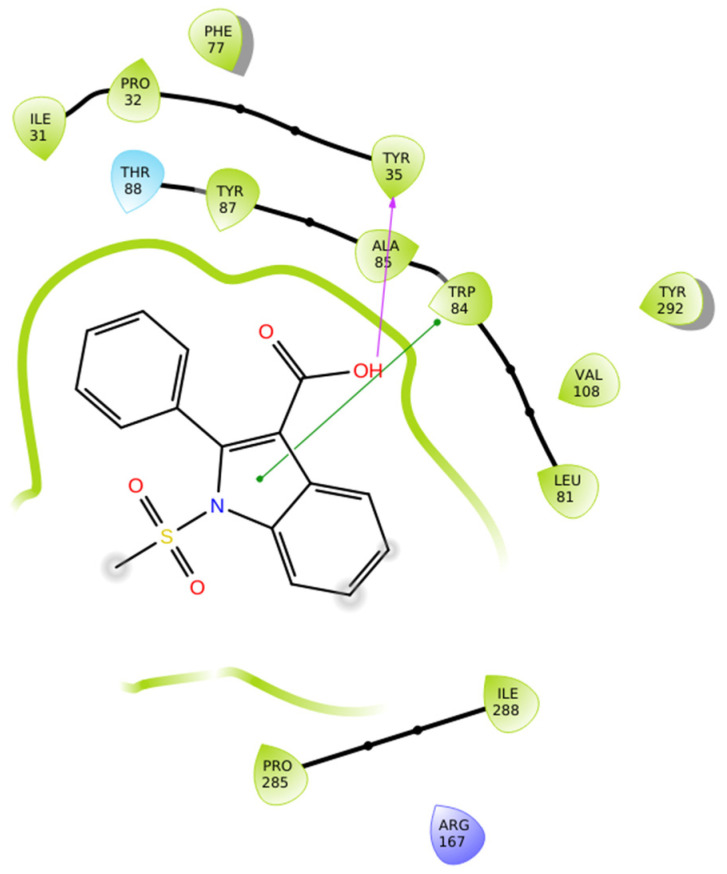
Two-dimensional ligand interactions’ diagram of compound **4a** bound to the AT1 receptor (PDB ID: 4ZUD). The hydroxyl group of the carboxylic moiety of indole forms a hydrogen bond with Tyr35. pi–pi stacking interaction occurs between Trp84 and the pyrrole ring of the indole moiety. Hydrophobic residues involved include Ile31, Pro32, Tyr35, Phe77, Tyr87, Ala85, Trp84, Leu81, Val108, Tyr292, Pro285, and Ile288. Thr88 contributes to polar stabilization, while Arg167 is identified as a positively charged residue within the binding pocket. The docking score (−10.068 kcal/mol) reflects a moderately favorable binding conformation.

Compound **4a** establishes a modest yet stable interactions’ network within the AT1 receptor (PDB ID: 4ZUD), primarily stabilized through aromatic and hydrophobic contacts (Figure 7). A single hydrogen bond is formed between the hydroxyl group of the carboxyl substituent of indole and Tyr35, which anchors the ligand in the binding pocket. pi–pi stacking between Trp84 and the pyrrole ring of the indole moiety enhances electronic stabilization and contributes to the overall binding affinity. A network of hydrophobic residues—comprising Ile31, Pro32, Tyr35, Phe77, Tyr87, Ala85, Trp84, Leu81, Val108, Tyr292, Pro285, and Ile288—reinforces the ligand’s spatial orientation through van der Waals interactions. Additional stabilization is provided by Thr88 (polar) and Arg167 (positively charged), which help maintain electrostatic balance within the binding environment (Figure 7). With a docking score of −10.068 kcal/mol, compound **4a** exhibits a stronger affinity than losartan (−9.739 kcal/mol) but a notably weaker one than **Hit 1** (−14.650 kcal/mol), compound **1a** (−14.782 kcal/mol), and compound **1g** (−12.894 kcal/mol). The relatively simpler interaction profile of **4a**—limited hydrogen bonding and a single pi–pi stacking contact—suggests that while the ligand achieves basic stabilization within the receptor pocket, it lacks the extensive multi-interaction framework observed in higher-affinity analogs. Structural optimization aimed at increasing polar or charged interactions could potentially enhance its binding performance toward the AT1 receptor.

**Figure 8:**
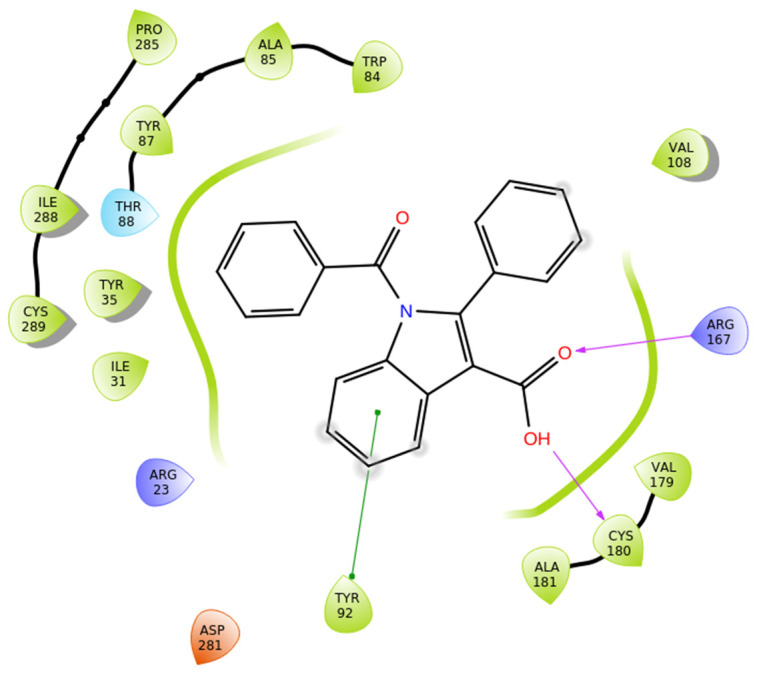
Two-dimensional ligand interactions’ diagram of compound **4g** bound to the AT1 receptor (PDB ID: 4ZUD). The ligand forms hydrogen bonds between the carbonyl oxygen of the carboxyl group of indole and Arg167, and between the hydroxyl group of the same carboxyl substituent and Cys180. A pi–pi stacking interaction is observed between Tyr92 and the phenyl ring of the indole moiety. Hydrophobic residues involved include Ile31, Tyr35, Tyr87, Ala85, Trp84, Val108, Val179, Cys180, Ala181, Tyr92, Pro285, Ile288, and Cys289. Thr88 contributes to polar stabilization, while Arg23 and Arg167 are positively charged residues, and Asp281 is negatively charged within the binding environment. The docking score (−10.430 kcal/mol) indicates a moderately strong and stable ligand–receptor interaction.

Compound **4g** exhibits a balanced interactions’ profile within the AT1 receptor (PDB ID: 4ZUD), stabilized by a combination of hydrogen bonding, pi–pi stacking, and hydrophobic interactions (Figure 8). The carboxyl group of the indole moiety engages in two key hydrogen bonds; the carbonyl oxygen interacts with Arg167, while the hydroxyl group forms an additional hydrogen bond with Cys180. These dual polar contacts provide essential anchoring within the receptor’s binding pocket. A pi–pi stacking interaction between Tyr92 and the phenyl moiety of the indole contributes to aromatic stabilization and proper ligand orientation. The surrounding hydrophobic residues—comprising Ile31, Tyr35, Tyr87, Ala85, Trp84, Val108, Val179, Cys180, Ala181, Tyr92, Pro285, Ile288, and Cys289—enhance van der Waals stabilization and reinforce the hydrophobic core of the complex. Polar Thr88 and charged residues (Arg23, Arg167, and Asp281) define an electrostatically balanced environment that further supports ligand accommodation (Figure 8). With a docking score of −10.430 kcal/mol, compound **4g** demonstrates a stronger affinity than losartan (−9.739 kcal/mol), yet lower than **Hit 1** (−14.650 kcal/mol), compound **1a** (−14.782 kcal/mol), and compound **1g** (−12.894 kcal/mol). The interaction pattern of **4g**, though less extensive than that of the high-affinity analogs, reveals a stable and well-oriented complex mediated by dual hydrogen bonding and rich hydrophobic contacts. These findings suggest that **4g** retains key pharmacophoric features necessary for AT1 receptor engagement, offering a promising scaffold for further optimization aimed at enhancing binding strength and selectivity.

**Figure 9:**
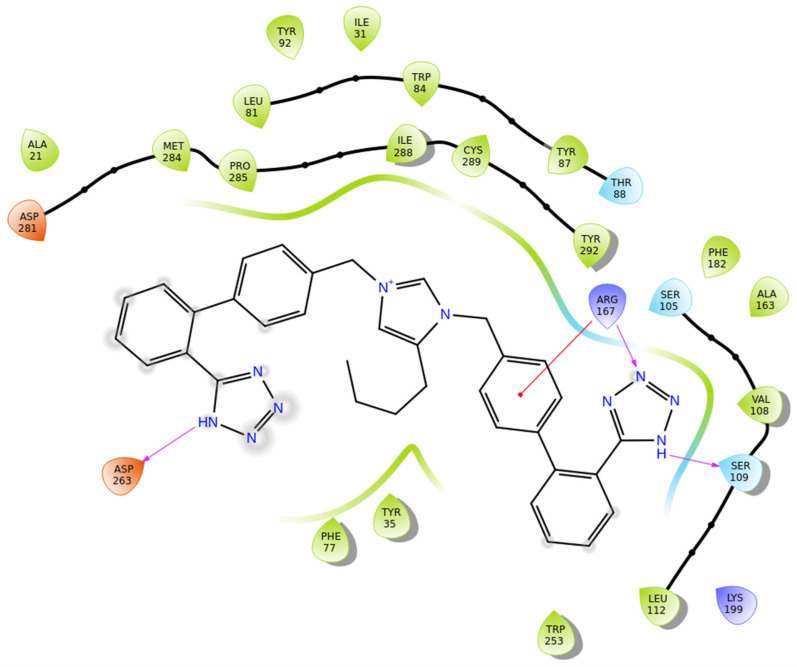
Two-dimensional ligand interactions’ diagram of compound **BisA** bound to the AT1 receptor (PDB ID: 4ZUD). The ligand forms hydrogen bonds between the nitrogen of the tetrazole group and Arg167, the amine moiety of the other tetrazole group and Asp263, as well as the tetrazole group and Ser109. A pi–cation interaction is observed between Arg167 and the phenyl moiety of the biphenyl group. Hydrophobic residues involved include Tyr92, Ile31, Leu81, Trp84, Tyr87, Ala21, Met284, Pro285, Ile288, Cys289, Tyr292, Phe182, Ala163, Val108, Leu112, Trp253, Tyr35, and Phe77. Polar Thr88, Ser105, and charged residue Lys199 define an electrostatically balanced environment that further supports ligand accommodation. The docking score (−10.057 kcal/mol) indicates a moderately strong and stable ligand–receptor interaction.

Compound **BisA** exhibits high affinity for the AT1 receptor (PDB ID: 4ZUD), stabilized by a combination of hydrogen bonding, pi–pi stacking, and hydrophobic interactions (Figure 9). The tetrazole group engages in three key hydrogen bonds; the nitrogen of the tetrazole group is a hydrogen bond acceptor, interacting with Arg167, while the amine moiety of the tetrazole group donates an additional hydrogen bond to Ser109. Moreover, the amine moiety of the other tetrazole group interacts through hydrogen bonding with Asp263. These dual polar contacts provide essential anchoring within the receptor’s binding pocket. A pi–cation interaction between Arg167 and the phenyl moiety of the biphenyl group contributes to aromatic stabilization. The surrounding hydrophobic residues—comprising Tyr92, Ile31, Leu81, Trp84, Tyr87, Ala21, Met284, Pro285, Ile288, Cys289, Tyr292, Phe182, Ala163, Val108, Leu112, Trp253, Tyr35, and Phe77—enhance van der Waals stabilization. Polar Thr88, Ser105, and charged residue Lys199 define an electrostatically balanced environment that further supports ligand accommodation (Figure 9). With a docking score of −10.057 kcal/mol, compound **BisA** demonstrates a stronger affinity than losartan (−9.739 kcal/mol), yet lower than all other studied compounds. The interaction pattern of **BisA**, extensive and comparable to the high-affinity analogs, reveals a stable and well-oriented complex mediated by dual hydrogen bonding and rich hydrophobic contacts. These findings suggest that **BisA** retains key pharmacophoric features necessary for AT1 receptor engagement, offering a promising scaffold for further optimization aimed at enhancing binding strength and selectivity.

**Figure 10:**
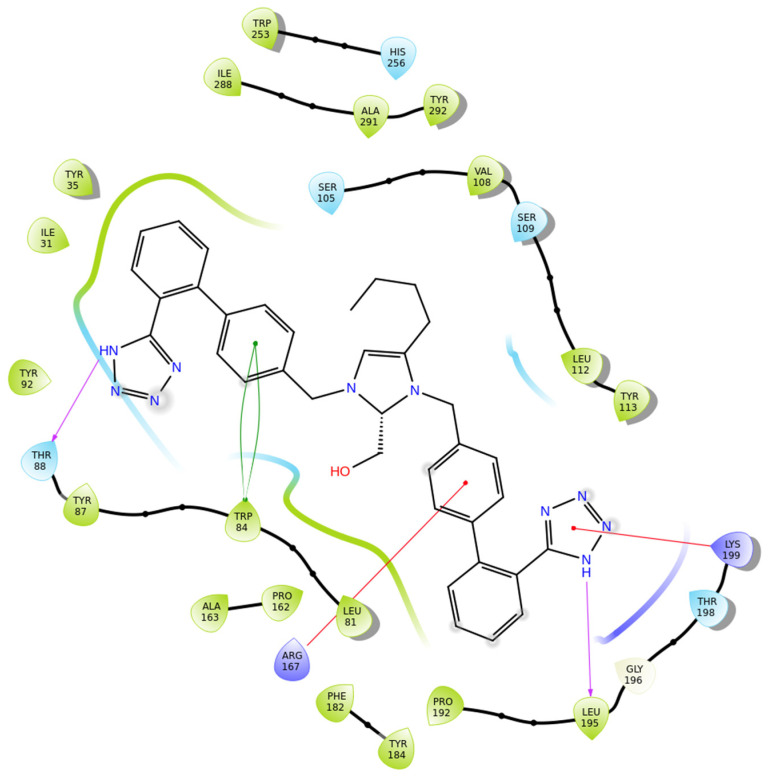
Two-dimensional ligand interactions’ diagram of compound **BisB** bound to the AT1 receptor (PDB ID: 4ZUD). The ligand forms hydrogen bonds between the nitrogen of the tetrazole group and Leu195 and between the amine moiety of the other tetrazole group and Thr88. pi–pi stacking between Trp84 and the phenyl ring of the biphenyl moiety enhances electronic stabilization and contributes to the overall binding affinity. pi–cation interactions are produced between Arg167 and the phenyl moiety of the biphenyl group and between Lys199 and the tetrazole ring, contributing to aromatic stabilization. Hydrophobic residues involved include Trp253, Ile288, Ala291, Tyr292, Val108, Leu112, Tyr113, Tyr35, Ile31, Tyr92, Tyr87, Ala163, Pro162, Leu81, Phe182, Tyr184, and Pro192. Polar His256, Ser105, Ser109, and Thr198 define an electrostatically balanced environment that further supports ligand accommodation. The docking score (−14.853 kcal/mol) indicates a very strong and stable ligand–receptor interaction.

Compound **BisB** showcases very high affinity for the AT1 receptor (PDB ID: 4ZUD), primarily stabilized through hydrogen bonding, aromatic, and hydrophobic contacts (Figure 10). Two hydrogen bonds are formed; one between the nitrogen of the tetrazole group and Leu195 and one between the amine moiety of the other tetrazole group and Thr88. pi–pi stacking between Trp84 and the phenyl ring of the biphenyl moiety enhances electronic stabilization and contributes to the overall binding affinity. Two pi–cation interactions are produced: one between Arg167 and the phenyl moiety of the biphenyl group and one between Lys199 and the tetrazole ring, contributing to aromatic stabilization. A network of hydrophobic residues—comprising Trp253, Ile288, Ala291, Tyr292, Val108, Leu112, Tyr113, Tyr35, Ile31, Tyr92, Tyr87, Ala163, Pro162, Leu81, Phe182, Tyr184, and Pro192—reinforces the ligand’s spatial orientation through van der Waals interactions. Additional stabilization is provided by polar residues His256, Ser105, Ser109, and Thr198, which help maintain electrostatic balance within the binding environment (Figure 10). With a docking score of −14.853 kcal/mol, compound **BisB** exhibits a stronger affinity than losartan (−9.739 kcal/mol) and all other inhibitors studied. The interaction pattern of BisB is extensive and comparable to the high-affinity analogs and reveals a highly stable and well-oriented complex mediated by hydrogen bonding and rich hydrophobic contacts. These results indicate that **BisB** preserves the essential pharmacophoric elements required for effective AT1 receptor interaction, positioning it as a promising structural framework for further studies.

Docking analysis of losartan, **Hit 1**, **1a**, **1g**, **4a**, **4g**, **BisA**, and **BisB** with the AT1 receptor (PDB ID: 4ZUD) revealed that binding affinity increases with the number and diversity of stabilizing interactions. Losartan showed moderate affinity (−9.739 kcal/mol) with limited hydrogen bonds and a single pi–pi stacking. **Hit 1** (−14.650 kcal/mol), **1a** (−14.782 kcal/mol), and **BisB** (−14.853 kcal/mol) displayed extensive hydrogen bonding, multiple pi–pi stacking interactions, and dense hydrophobic contacts, resulting in highly stable complexes. Compound **1g** (−12.894 kcal/mol) maintained strong affinity through dual hydrogen bonds and rich hydrophobic interactions, while **4a** (−10.068 kcal/mol) and **4g** (−10.430 kcal/mol) exhibited simpler yet stable binding patterns. **BisA** (−10.057 kcal/mol), on the other hand, although showcasing a complex binding pattern, does not exhibit the low binding energy that **BisA** exhibits. Overall, data indicate that from the 2020–2024 studied compounds, compound **1a** forms the most favorable and energetically stable complex with the AT1 receptor, combining an extensive interaction network with optimal geometric complementarity. On the other hand, **BisB** shows the overall best binding affinity for the AT1 receptor. Table 1 summarizes the computational docking results alongside the IC_50_ values of the chosen compounds.

It should be noted that while docking studies give satisfactory predictions regarding the overall binding affinity of a ligand to a protein, they do not always correlate exactly to the actual inhibitory activity of the molecules in vitro. That is due to many different factors contributing to this discrepancy. For example, **Hit 1** showcases one of the lowest binding scores (−14.650 kcal/mol), yet its IC_50_ value (19.6 nM) is only slightly lower than that of Losartan (20 nM). That is because larger molecules tend to form more interactions with more amino acids of the binding pocket. Also, the IC_50_ value of a ligand is not always specific in regard to the affinity towards a target, but other physicochemical characteristics, such as its ability to penetrate lipid bilayers, play a pivotal role in its overall inhibitory potency as well.

### 4.2. Structural Analysis

The molecules studied via computational chemistry can now be studied based on the developed ligand–receptor interactions. Regarding the molecules from 2020 to 2024, compounds **1a** and **Hit 1** showcase the most negative docking scores of −14.782 and −14.650 kcal/mol, respectively. The reason why these molecular complexes are characterized as more stable and have such low binding energies is due to their extended hydrogen bond interactions. Specifically, both compound **1a** and **Hit 1** develop hydrogen bonds with amino acid Arg167, while they differentiate on all other types of amino acid residues they interact with. Compound **1a** develops hydrogen bonds with the following amino acids: His256, Arg167, Tyr35, Tyr87, and Cys180, while **Hit 1** interacts with Thr88, Tyr92, and Arg167 via hydrogen bonding. This means that compound **1a** interacts with five amino acid residues via hydrogen bonding, while **Hit 1** interacts with three through the same kind of interaction. All other compounds studied (losartan, **1g**, **4a**, and **4g**) develop fewer than three hydrogen bonds with the active site of the AT1 receptor. Thus, compounds **1a** and **Hit 1** score lower in binding energy values (Table 2).

Regarding the other compounds, they can be ranked in increasing order of binding energy as follows: **1g** (−12.894 kcal/mol) < **4g** (−10.430 kcal/mol) < **4a** (−10.068 kcal/mol) < losartan (−9.739 kcal/mol). This is due to the kind and amount of interactions they develop with amino acid residues. Specifically, compound **1g** develops hydrogen bond interactions with two amino acid residues (Arg167 and Cys180) and pi–pi interactions with two amino acid residues (Trp84 and Tyr292). On the other hand, compound **4g** develops hydrogen bond interactions with two amino acid residues (Arg167 and Cys180) and pi–pi interactions with only one amino acid residue (Tyr92). Finally, compound **4a** develops hydrogen bond interactions with only one amino acid residue (Tyr35) and pi–pi interactions with only one amino acid residue (Trp84). Losartan develops hydrogen bond interactions with two amino acid residues (Thr88 and Tyr35) and pi–pi interactions with one amino acid residue (Tyr92), as well as utilizes Arg167 as a positively charged amino acid residue for the stabilization of the binding pocket. Nevertheless, this does not translate directly to a higher binding energy compared to compound **4a**, nor is it of the same magnitude as compound **4g**. That means that the energy is not correlated only to the amount of amino acid residues interacted with the compound, either via hydrogen bonding or pi–pi interactions, but it is also amino acid specific. In a closer look, we can observe that between compounds **4a** and losartan, which both develop hydrogen bonds with Tyr35, the interaction of compound **4a** with Trp84 via pi–pi stacking has a greater impact on the overall energy of the complex, stabilizing it more (−10.068 kcal/mol) in comparison to losartan (−9.739 kcal/mol), which interacts with Thr88 via hydrogen bonding (Table 2).

Based on these observations, newly synthesized compounds should be focused on having the appropriate scaffold in order to interact with amino acid residues Arg167, Tyr87, Cys180, and His256 via hydrogen bonding and with Trp84 and Tyr292 via pi–pi interactions.

On the other hand, compound **BisA** interacts in a different way with the AT1 receptor, while BisB has an interaction profile closely resembling the one for the previously mentioned compounds. Interaction with Arg167 plays a crucial role in binding energy via the formation of either hydrogen bonds or pi–cation interactions. **BisA** forms hydrogen bonds with Ser109 and Asp263 and interacts in two ways with Arg167: through hydrogen bonding and pi–cation interactions. **BisB** interacts via hydrogen bonding with Thr88 and Leu195, through pi–pi stacking with Trp84, and via pi–cation interactions with Arg167 and Lys199. The very low binding energy of **BisB** (−14.853 kcal/mol) indicates that amino acids Arg167, Thr88, and Trp84 do play an important role in overall affinity for the target (Table 2).

### 4.3. Pharmacophore Model

**Figure 11:**
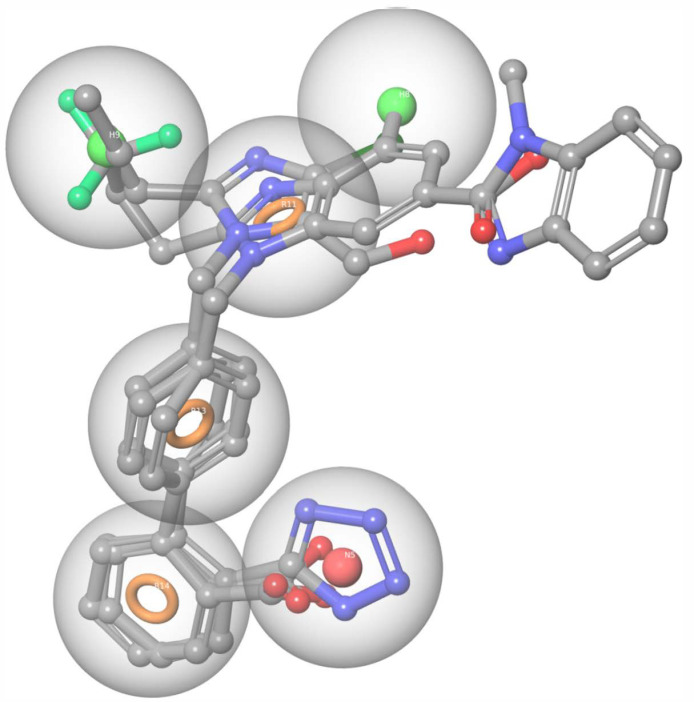
Pharmacophore model developed using Maestro 2021-2 software. The important pharmacophore moieties were derived from the superimposition of the active compounds from Table 1. Newly discovered compounds should possess these structural elements in order to exhibit potent inhibitory activity against AT1R. Specifically, they should contain three aromatic rings (orange circles), one acidic point (red sphere), and two hydrophobic positions (green spheres).

Furthermore, a pharmacophore model was developed utilizing phase software as implemented on Maestro 2021-2. The dataset was divided into active compounds (Table 1) and inactive compounds, which were selected based on compounds with lower affinities for the AT1R from the bibliography [107,108,109,110,112]. The phase algorithm resulted in eleven hypotheses. The most dominant hypothesis is presented in Figure 11. The hypothesis consists of three aromatic rings (orange circles), one acidic point (red sphere), and two hydrophobic positions (green spheres), which constitute the important pharmacophore structures that all AT1R potent inhibitors should possess.

## 5. Conclusions

The renin–angiotensin–aldosterone system (RAAS) plays a crucial role in regulating blood pressure and maintaining fluid and electrolyte balance. It also drives significant structural and functional remodeling within the cardiovascular system, including the heart and blood vessels. Consequently, the RAAS serves as a central therapeutic target in a range of chronic cardiovascular disorders, including arterial hypertension (AH) and heart failure (HF). In this review, we aimed to describe the RAAS with particular focus on the structural characteristics of the angiotensin II type 1 receptor (AT1R) and its role in the pathophysiology of hypertension. Key biomolecules of the system are extensively described. The structure and function of the AT1 receptor are also presented in detail, and its active site is defined. Consequently, a bibliographic review takes place for the years 2020–2024, showcasing the developments of AT1R antagonists. These compounds can be divided into three major classes: large peptide-like molecules, small non-peptide molecules, and sartan derivatives. This showcases the fact that sartans and sartan derivatives are not the only possible ways to create potent inhibitors of the AT1 receptor. The fact that both large peptide-like molecules and small non-peptide molecules show these low IC_50_ values is a major implication that the chemical space for the development of AT1R antagonists is not yet well-explored and is full of potential for rational drug design. An attempt is made to determine, with the help of computational chemistry tools, the key interactions of the molecules found and described in this review from the literature, as well as to draw conclusions regarding the reasons why these different molecules exhibit different binding energies. From structural analysis, it was deduced that the key amino acid residues a novel molecule should interact with in order to showcase very low binding energies are Arg167, Tyr87, Cys180, and His256 via hydrogen bonding and Trp84 and Tyr292 via pi–pi interactions. Further NMR experiments should, of course, be performed to verify these observations, but it is clear where the scientific community should turn its attention for the development of more potent inhibitors of the AT1 receptor. Finally, a pharmacophore model was also developed using Maestro software. The important pharmacophore moieties that newly discovered compounds should possess in order to exhibit potent inhibitory activity against AT1R are as follows: three aromatic rings, one acidic point, and two hydrophobic positions. We believe that our provided model can enhance drug design and development, assisting medicinal chemists in the creation of more potent antihypertensive medications.

## Figures and Tables

**Table 2:** Comparative data of the formed ligand–protein interactions established between the studied compounds (2020–2024) and the AT1 receptor. Representative bisartan compounds and their interactions with the AT1 receptor are included as well. The compounds have been placed top to bottom from the lowest to the highest binding score.

Name	Amino Acid Residues (Type of Interaction)									
**1a**		Tyr35(H)		Arg167(H)		His256(H)	Cys180(H)	Tyr87(H)		
**Hit 1**	Thr88(H)		Tyr92(H,pi)	Arg167(H)	Trp84(pi)					
**1g**				Arg167(H)			Cys180(H)		Trp84(pi)	Tyr292(pi)
**4g**			Tyr92(pi)	Arg167(H)			Cys180(H)			
**4a**		Tyr35(H)							Trp84(pi)	
**Losartan**	Thr88(H)	Tyr35(H)	Tyr92(pi)							
**BisB**	Thr88(H)			Arg167(pic)				Leu195(H)	Trp84(pi)	Lys199(pic)
**BisA**				Arg167(H,pic)		Asp263(H)	Ser109(H)			

The symbol H in parentheses implies hydrogen bonds between the specified amino acid and the compound. The symbol pi in parentheses implies the pi–pi interactions between the specified amino acid and the compound. The symbol pic in parentheses implies the pi–cation interactions between the specified amino acid and the compound.

## Data Availability

No new data were created or analyzed in this study. Data sharing is not applicable to this article.

## Abbreviations

The following abbreviations are used in this manuscript:

ACE	Angiotensin-converting enzyme
ARBs	Angiotensin II receptor blockers
AT1R	Angiotensin II Type 1 receptor
ECF	Extracellular fluid
GI	Gastrointestinal
RAAS	Renin–Angiotensin–Aldosterone system
Ang 1–7	Angiotensin 1–7
Ang 1–9	Angiotensin 1–9
ALA	Alamandine
AGT	Angiotensinogen
Ang I	Angiotensin I
Ang II	Angiotensin II
Ang III	Angiotensin III
Ang IV	Angiotensin IV
ACE1	Angiotensin Converting Enzyme Type 1
AT2R	Angiotensin II Type 2 receptor
MRs	Mineralocorticoid receptors
ROS	Reactive oxygen species
AP-A	Aminopeptidase-A
AP-N	Aminopeptidase-N
IRAP	Insulin-regulated aminopeptidase
RAS	Renin–Angiotensin system
Ang A	Angiotensin A
NEP	Neprilysin
MasR	MAS proto-oncogene receptors
ACE2	Angiotensin Converting Enzyme Type II
GPCRs	G protein-coupled receptors
PLC	Phospholipase C
PKC	Protein kinase C
ECLs	Extracellular loops
TMDs	Transmembrane domains
IP3	Inositol trisphosphate
PDB	Protein Data Bank
SAR	Structure–Activity Relationship
